# The effectiveness of using recombinant human bone morphogenetic protein-2 in the reconstruction of alveolar clefts: a meta-analysis

**DOI:** 10.3389/froh.2026.1786495

**Published:** 2026-06-16

**Authors:** Hongtu Xu, Zhina Liu

**Affiliations:** Shangrao People's Hospital, Shangrao, Jiangxi Province, China

**Keywords:** alveolar bone grafting, alveolar ridge crack, cleft lip, cleft palate, recombinant human bone morphogenetic protein-2t

## Abstract

**Objectives:**

The purpose of this study was to examine the available evidence on the impact of using recombinant human bone morphogenetic protein-2 (rhBMP-2) in the reconstruction of alveolar clefts in terms of bone formation rate, bone volume, bone density, and postoperative complications.

**Methods:**

The PubMed, Embase, Web of Science, Scopus, and Cochrane databases were searched until April 2024. The outcomes included bone formation rate, bone volume, bone density, and postoperative complications. Studies comparing autologous iliac bone and rhBMP-2 for osteogenesis in patients with alveolar clefts were included.

**Results:**

Six studies were included. Treatment of an alveolar cleft with autologous iliac bone grafting resulted in higher bone formation, with statistically significant results after a 6-month follow-up period (MD −14.54; 95% CI −21.58 to −7.49; *p* < 0.0001). Furthermore, at 6 months (MD 186.60; 95% CI 33.26–339.94; *p* = 0.02) and 12 months (MD 188.70; 95% CI 25.13–352.27; *p* = 0.02), bone density was lower and the difference was statistically significant. The pooled data indicate that autologous bone graft resulted in statistically significantly higher bone volume (MD −163.09; 95% CI −313.45 to −12.74; *p* = 0.03). Regarding postoperative complications, no differences were found between the two groups (MD 0.67; 95% CI 0.25–1.78; *p* = 0.42).

**Conclusion:**

In this study, the application of rhBMP-2 showed no obvious advantage in the reconstruction of alveolar clefts in terms of bone volume and bone formation rate and may result in significant harm. However, further clinical research is required.

**Systematic Review Registration:**

https://www.crd.york.ac.uk/PROSPERO/reviewcoversheet

## Background

The treatment of the alveolar cleft is a very important part of the sequential treatment of patients with a cleft lip and palate. Secondary alveolar bone graft (ABG) has become the gold standard in alveolar cleft (1), and the advantages of bone grafting include stabilizing the maxillary arch and promoting tooth eruption (2, 3). Surgeons are always searching for suitable alternatives to bone grafting because autologous bone grafting has donor complications and the autologous bone mass is not sufficient when the defect is large (4, 5).

An ideal bone graft material should be non-immune, contain growth factors that promote rapid bone formation and revascularization, maintain space for new bone infiltration, and be readily available in the clinical setting (6). Bone morphogenetic protein (BMP) has been found to have the effect of promoting bone regeneration when used with drug carriers (7). In particular, BMP-2 can act during the entire osteogenesis stage from mesenchymal stem cell osteoprogenitor cells to osteoblasts, promoting new bone formation (6, 8). BMP-2 also shows potential in bone regeneration, with applications such as alveolar bone preservation, sinus enlargement, bone augmentation, and periodontal recovery (9–12). With the increase in the clinical use of BMP-2, well-documented side effects have emerged. These include postoperative inflammation and associated adverse effects, ectopic bone formation, osteoclast-mediated bone resorption, and inappropriate adipogenesis (13). Because the use of recombinant human BMP-2 (rhBMP-2) in alveolar fissures has been controversial (14), its use in alveolar bone grafting has never produced consistent and reliable results, even though rhBMP-2 has been approved as an alternative to autologous bone grafting (15).

Thus, it is necessary to assess the available evidence for this procedure and identify best practices and high-quality primary studies on this topic.

## Materials and methods

### Registration

This meta-analysis followed the PRISMA Guidelines checklist. The protocol was registered at PROSPERO (CRD42023397628), the international prospective register of systematic reviews.

### Eligibility criteria

Randomized controlled trials (RCTs) related to the application of bone morphogenetic protein in alveolar cleft bone grafting were included. The inclusion criteria were patients with the diagnosis of cleft lip and palate (CLP), radiographic evaluation of the cleft area, age 7–15 years, and at least a 3-month follow-up. Exclusion criteria were an edentulous maxilla, an atypical or non-described cleft diagnosis, and associated syndrome conditions.

### Literature retrieval strategies

The meta-analysis was conducted according to the Preferred Reporting Items for Systematic Reviews and Meta-Analyses (PRISMA) guidelines (16). The search strategy in this study was conducted according to the PICOS (population/patient intervention comparison/control outcome study design) model as follows: P, alveolar cleft patients; I, recombinant human bone morphogenetic protein-2; C, autologous bone graft; O, bone formation rate, bone volume, bone density, and number of complications; S, clinical control study. Moreover, the search terms were alveolar cleft, bone morphogenetic protein, and autologous bone. Relevant studies published before February 2023 were searched for in PubMed, The Cochrane Library, Embase, Web of Science, and Scopus.

### Search methodology

There was no language restriction. An electronic search was independently conducted by two authors (HTX and ZNL) in PubMed/MEDLINE, using the Medical Subject Heading terms combined with free words, including “maxillary,” “alveolar,” “bone transplantation,” “cleft reconstruction,” “cleft defect,” “premaxillary cleft,” “cleft palate,” “cleft lip,” “alveolar Bone grafting,” “recombinant proteins,” “transforming growth factor beta,” and “bone morphogenetic protein 2” for studies published until February 2023. A similar search was conducted in the Cochrane Central Register of Controlled Trials (CONTROL) via the Cochrane Library, EMBASE via Ovid, Web of Science, and Scopus. To search for any additional research relevant to this review, a secondary search was undertaken by perusing the reference lists of the articles that met the inclusion criteria. The two reviewers then independently reviewed the full-text copies, and any disagreements on the studies’ eligibility were resolved through a consensus discussion. In the second selection round, studies that did not meet the inclusion criteria were eliminated.

### Study selection

The search results were exported to EndNote X9 software to remove any duplicates. The two reviewers (HTX and ZNL) independently and randomly examined the titles and abstracts of all the studies by examining the titles, abstracts, and keywords. The full texts of all the apparently relevant studies or those for which there were insufficient data in the title and abstract to make a clear decision were obtained. The full-text articles were then independently assessed by two reviewers, and any disagreement on the eligibility of included studies was resolved through a consensus discussion. Studies that did not match the inclusion criteria in this third selection phase were excluded (Table 1).

**Table 1:** Characteristics of the excluded studies.

Study	Reason for exclusion
Boyne et al., 2005	No control group
Alonso et al., 2010	Used the latest study
Alonso et al., 2014	No information about the bone volume, density, formation rate, or morbidity
Ayoub et al., 2016	No control group
Nam et al., 2017	The control group was not autogenous

### Data collection process

The data from the included studies were retrieved by the two reviewers independently. Data on the study setting, characteristics of the study sample (such as age and patient type), graft sources, and results were extracted. The sources of funding for any included studies were noted if they were indicated. Table 2 shows the extracted data from the included studies.

**Table 2:** Characteristics of the included studies.

Author (year)	Study design, type of patient (duration)	Type of BMP (concentration and dose)	Type of autogenous bone	Age (mean/range)	Fund	Outcome measuring method	Study group (n)	Outcomes
Herford et al., 2007 (23)	RCT, unilateral cleft premaxilla (4 months)	rhBMP-2 (1.05 mg/mL, 4.2 mg)	Autogenous iliac crest bone	7–11	No	CT	EG: rhBMP-2 (*n* = 10) CG: autogenous bone graft (*n* = 2)	Bone graft filling rate (4 months), complications (swelling)
Dickinson et al., 2008 (22)	RCT, UCLP (12 months)	BMP-2 (1.5 mg/mL, not reported)	Autogenous iliac crest bone	Not reported	Yes	NewTom	EG: BMP-2 (*n* = 9) CG: autogenous bone graft (*n* = 12)	Complications (loss of bone graft and oronasal fistula)
Canan et al., 2012 (21)	RCT, cleft and alveolar defect (12 months)	rhBMP-2 (1.5 mg/mL, 3.2 mg–4.2 mg)	Autogenous iliac crest bone	8–15	Yes	CT	EG: rhBMP-2 (*n* = 6) CG: autogenous bone graft (*n* = 6)	Bone graft filling rate, bone volume, bone density (3, 6, and 12 months)
Neovius et al., 2013 (25)	RCT, unilateral alveolar cleft defect (6 months)	BMP-2 (0.05 mg/mL, 0.25 mg/mL, not reported)	Autogenous iliac crest bone	8–12	Yes	CT	EG: BMP-2 (*n* = 3,) CG: autogenous bone graft (*n* = 4)	Bone volume (3 months), complications (swelling)
Liang et al., 2017 (24)	RCT, UCLP (9 months)	rhBMP-2 (1.5 mg/mL, 2.1 mg)	Autogenous iliac crest bone	Not reported	No	CBCT	EG: rhBMP-2 (*n* = 21) CG: autogenous bone graft (*n* = 14)	Complications (graft exposure)
Tanikawa et al., 2020 (26)	RCT, UCLP (12 months)	rhBMP-2 (1.5 mg/mL, 3.2 mg–4.2 mg)	Autogenous iliac crest bone	8–12	No	CT	EG: rhBMP-2 (*n* = 8) CG: autogenous bone graft (*n* = 8)	Bone graft filling rate (6 months, 12 months), complications (swelling)

UCLP, unilateral cleft lip and palate; rhBMP-2, recombinant human bone morphogenetic protein-2; BMP-2, bone morphogenetic protein-2; RCT, randomized controlled trial; CT, computed tomography; CBCT, cone beam computed tomography; NewTom, three-dimensional, panorex, periapical films.

### Outcomes

The primary outcomes were radiographic assessment of bone graft volume, radiographic assessment of the alveolar bone density of the grafted area, and complications after surgery. Considering that alveolar bone grafts are impacted by a number of factors and the initial conditions in the included studies varied, bone formation rate, bone density, and bone volume were used to assess bone quality and quantity, and the number of postoperative complications was recorded. The bone formation rate (BF%) was calculated as follows: [the postoperative formed bone volume (FV)/the volume of the actual bone graft (AV)] 100% = BF% (17). The secondary outcomes were length and cost of hospital stay. The effects of rhBMP-2 on alveolar bone grafting were compared independently. Participant characteristics, such as average age and size of bone insufficiency, were also recorded.

### Risk of bias in individual studies

**Figure 1:**
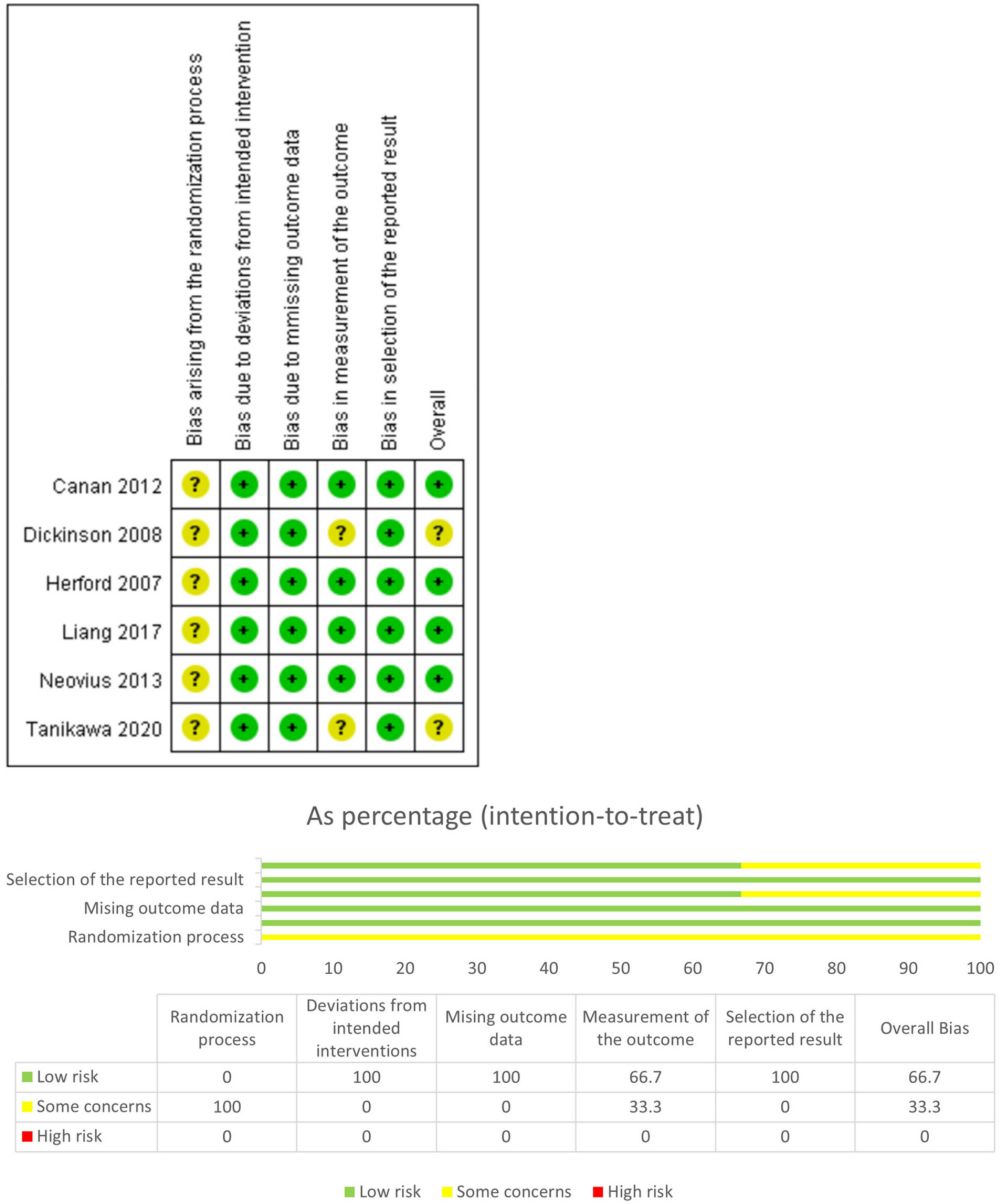
Assessment of the risk of bias in the included studies.

The assessment of the risk of bias in the included studies was undertaken independently by two reviewers. The Cochrane Collaboration's tool for assessing risk of bias was used (18). Disagreements in the classification were resolved through discussion. The following domains were assessed as having “low,” “high,” or “unclear” risk of bias: bias arising from the randomization process, bias due to deviations from the intended intervention, bias due to missing outcome data, bias in measurement of the outcome, bias in the selection of the reported results, and overall bias (Figure 1).

### Summary measures

For descriptive continuous data (bone formation rate, bone density, and bone volume), mean, standard deviation (SD), sample size, weight, and mean differences are reported. For descriptive count data (complications after surgery), events, total number, weight, and odds ratio are reported. A *p*-value of less than 0.05 was considered statistically significant, indicating there was a statistically significant difference between the control and intervention groups. When the homogeneity between the studies was good (*P* ≥ 0.10 and I^2^ ≤ 50%), a fixed-effect model was used for data merging. When *P* < 0.10 and I^2^ > 50%, random-effects models were used. Data analyses were performed using Review Manager 5.3 (Cochrane IMS, Copenhagen, Denmark) (19). When there was high homogeneity, a sensitivity analysis was performed.

### Risk of bias across studies

The Grading of Recommendation, Assessment, Development, and Evaluation (GRADE) instrument assessed the evidence quality and grading of recommendation strength of the included studies in the quantitative synthesis and meta-analysis (20). This assessment was based on considerations such as study design, consistency, directness, precision, publication bias, and other aspects reported by the studies included in this systematic review. The quality of the evidence was characterized as high, moderate, low, or very low and was assessed using tools from the http://gradepro.org website.

## Results

### Study selection

**Figure 2:**
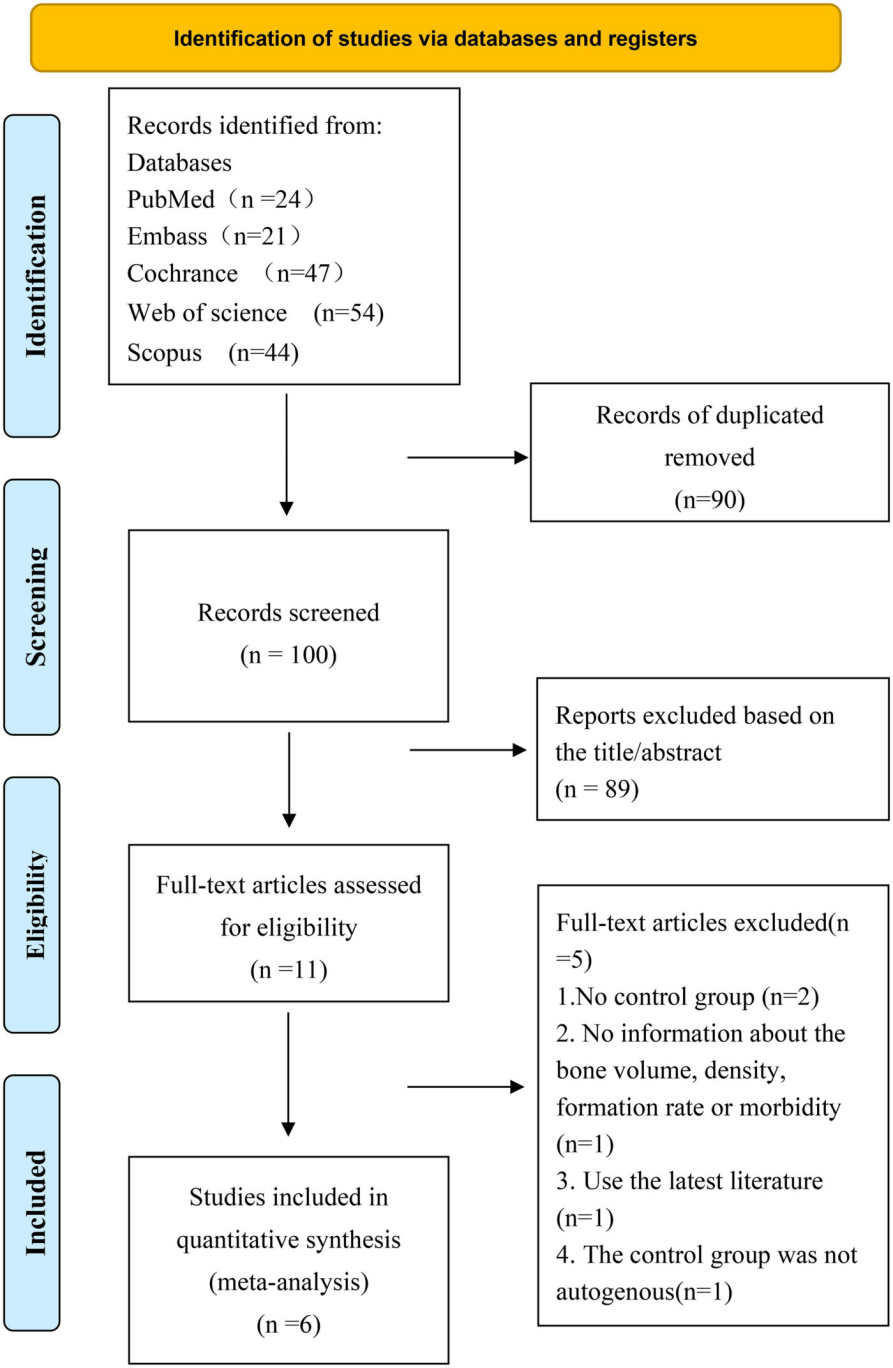
Flow diagram (additional material).

In the literature search, 190 publications were identified, of which 179 were excluded after reviewing the titles and abstracts. The full texts were obtained for the remaining 11 publications. After screening the full texts, five studies were excluded. Therefore, only six randomized controlled trials fulfilled all the inclusion criteria (21–26). Tables 1 and 2 present the details of the included studies examined and the reasons for inclusion and exclusion. The study identification process is illustrated in Figure 2.

### Study characteristics

A total of 103 patients with a unilateral cleft lip and palate were included, of which 57 underwent a rhBMP-2 graft and 46 patients underwent an autologous bone graft. Six primary articles published between 2000 and 2022 were found. All the studies were randomized clinical trials. The number of patients treated with rhBMP-2 ranged between 3 and 21, with an average of 9.5 patients. The average age of patients was 10 years in the majority of the studies. Of the six selected studies, two were conducted in Brazil, three in the USA, and one in Sweden. In all the articles, the use of rhBMP-2 for alveolar cleft reconstruction was compared to autologous bone grafts. In five of these studies, rhBMP-2 delivered by a collagen sponge carrier in the intervention group and the control group received a traditional iliac crest and cancellous bone graft. RhBMP-2 was delivered by a hydrogel carrier in the intervention group in only one study. The concentration of rhBMP-2 used was not uniform, and two studies had concentrations below 1.5 mg/mL. Only one trial found no complications, whereas the others found complications such as loss of bone graft, oronasal fistula, graft exposure, and swelling. Follow-up was carried out over a minimum of 4 months and a maximum of 12 months. Three studies were funded and the remaining studies were not funded.

None of the studies was assessed as having a “high” risk of bias. Details of the risk of bias assessment are as follows:

1. Bias arising from the randomization process: All of the included studies reported random allocation, but none reported sequence generation details. Thus, sequence generation was unclear.
2. Bias due to deviations from the intended intervention: None of the included studies clearly described any deviations from the intended intervention.
3. Bias due to missing outcome data: None of the patients from these studies were lost to follow-up, so the outcome data were considered to have a low risk of bias.
4. Bias in the measurement of the outcome: In two of the studies, the measurement of the resulting data was not blind, so they were considered to have “unclear” risk of bias, while the rest had a low risk of bias.
5. Bias in the selection of the reported result: There were no reported dropouts in any of the studies. Negative results were also reported.
6. Overall: Two of the studies were assessed to have an “unclear” risk of bias.

### Synthesis of results

The included studies compared the use of recombinant human bone morphogenetic protein-2 with traditional autologous bone grafting. They radiologically evaluated bone formation rate, bone density, and bone volume and recorded complications after surgery. CT and cone beam CT (CBCT) were used for radiological assessment (Table 2). Radiological evaluations were obtained preoperatively, after 3 or 4 months, and after 6 months or after 1 year of follow-up. No data were reported regarding the length of hospital stay and cost.

### Bone formation rate

**Figure 3:**
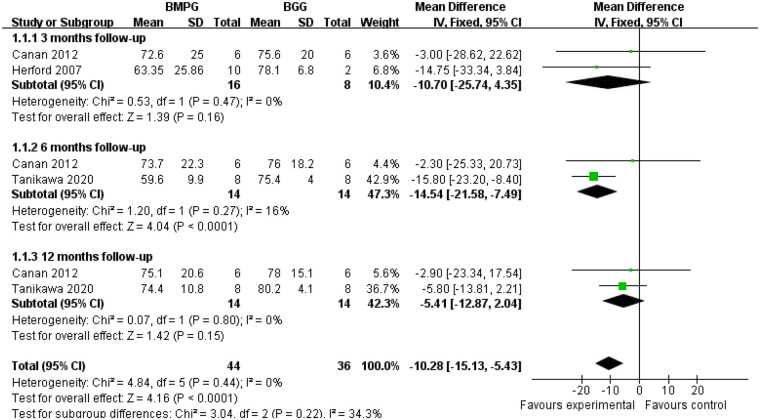
Forest plot of the mean difference (MD) estimate of the bone formation ratio after alveolar cleft reconstruction that involves autologous bone grafting, with the use of recombinant human bone morphogenetic protein-2 (rhBMP-2) as the control intervention. The bone formation ratio (BF%) was calculated as follows: [the postoperative formed bone volume (FV)/the volume of the actual bone graft (AV)] 100% = BF%.

Canan et al. and Herford et al. assessed bone formation rate using CT after 3 months. No statistically significant difference was noted (MD −10.07; 95% CI −25.74–4.35; *p* = 0.16) (Figure 3). Canan et al. and Tanikawa et al. assessed bone formation rate using CT after 6 months and 12 months, respectively. Autologous bone graft resulted in statistically significantly higher bone formation after a 6-month follow-up period (MD −14.54; 95% CI −21.58 to −7.49; *p* < 0.0001) (Figure 3). After 12 months of follow-up, this difference disappeared and no statistically significant difference was noted (MD −5.41; 95% CI −12.87–2.04; *p* = 0.15) (Figure 3). The heterogeneity may have been caused by differences in operation procedures, the outcome measuring method, age of the patients, and the preoperative defect volume. Because there were two included studies in each group, a sensitivity analysis was not required.

### Bone graft volume

**Figure 4:**
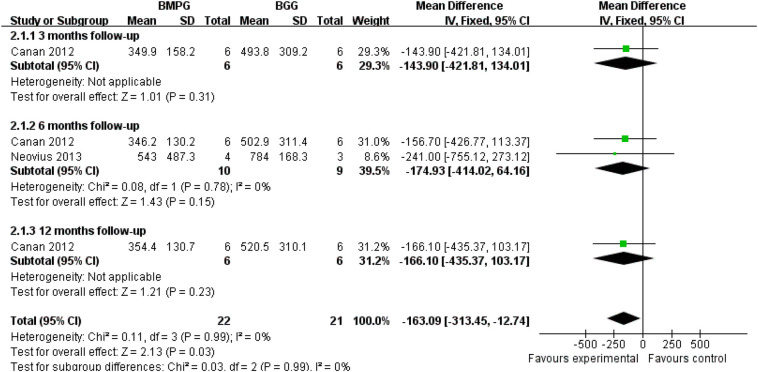
Forest plot of the mean difference (MD) estimate of the change in bone volume after alveolar cleft reconstruction that involves autologous bone grafting, with the use of recombinant human bone morphogenetic protein-2 (rhBMP-2) as the control intervention. The analysis over time shows the difference between the groups (fixed-effects model).

**Figure 5:**
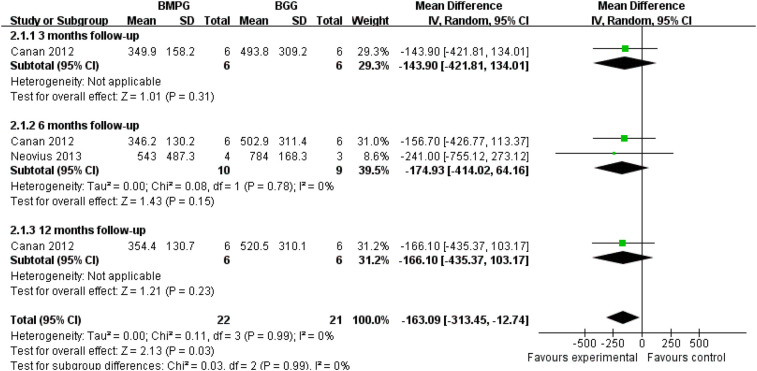
Forest plot for the sensitivity analysis of bone volume changes using a random-effects model.

Canan et al. evaluated bone volume using CT after 3 months. No statistically significant difference was noted (MD −143.90; 95% CI −421.81–134.01; *p* = 0.31) (Figure 4). Canan et al. and Neovius et al. assessed bone volume using CT after 6 months. No statistically significant difference was noted (MD −174.93; 95% CI −414.02–64.16; *p* = 0.15) (Figure 4). Moreover, Canan et al. assessed bone volume using CT after 12 months and there was no significant difference between the two groups (MD −166.10; 95% CI −435.37–103.17; *p* = 0.23) (Figure 4). A sensitivity analysis was necessary given the small sample sizes of the included studies, especially as only one study had 3 months and 12 months of follow-up. We pooled the data using a fixed-effects model, but we also analyzed the data using the random-effects model to ensure robustness of the model chosen and susceptibility to outliers. The two models showed consistent and stable results (Figure 5).

### Bone graft density

**Figure 6:**
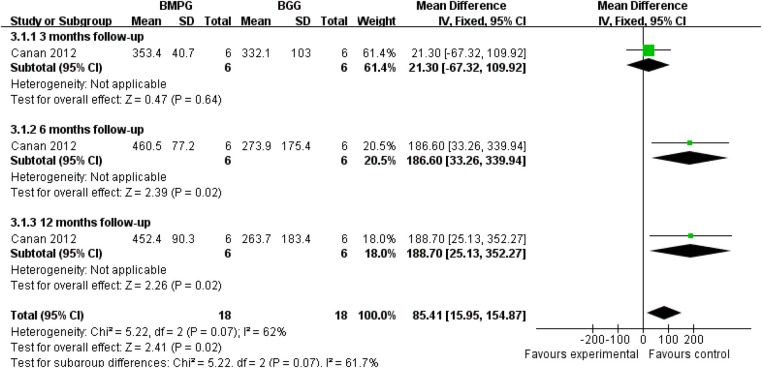
Forest plot of the mean difference (MD) estimate of the change in bone density after alveolar cleft reconstruction that involves autologous bone grafting, with the use of recombinant human bone morphogenetic protein-2 (rhBMP-2) as the control intervention. The analysis over time shows the difference between the groups (Hounsfield unit; fixed-effects model).

**Figure 7:**
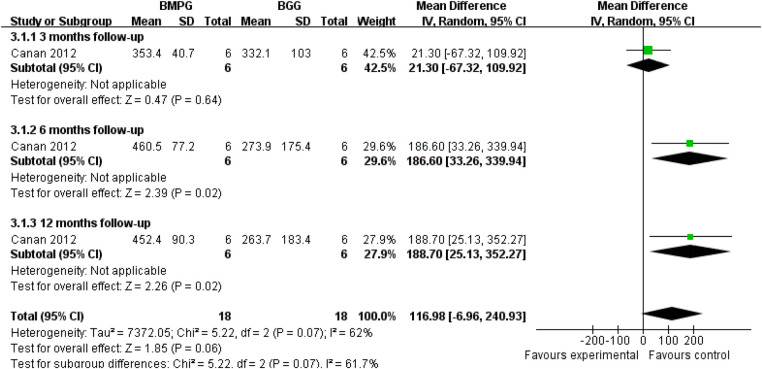
Forest plot for the sensitivity analysis of bone density changes using a random-effects model.

Canan et al. assessed bone density using radiological evaluations, which verified rhBMP-2's effect in alveolar cleft grafting. The meta-analysis assessed the effect of rhBMP-2 using a fixed-effects model. The follow-up time was different in each study; thus, a sectionalized analysis was performed to evaluate the influence of follow-up time on the meta-analysis outcome. No statistically significant difference was noted after 3 months (MD 21.30; 95% CI −67.32–109.92; *p* = 0.64) (Figure 6). However, statistically significant differences in bone density were noted after 6 months (MD 186.60; 95% CI 33.26–339.94; *p* = 0.02) and 12 months (MD 188.70; 95% CI 25.13–352.27; *p* = 0.02) (Figure 6). Given the small number of studies, the random-effects model was generally more appropriate. However, it is worth mentioning that the pooled results of the studies ranged from statistically significant (fixed-effects model) (MD 85.41; 95% CI 15.95–154.87; *p* = 0.02) (Figure 6) to not statistically significant (random-effects model) (MD 116.98; 95% CI −6.96–240.93; *p* = 0.06) (Figure 7).

### Complications after surgery

**Figure 8:**
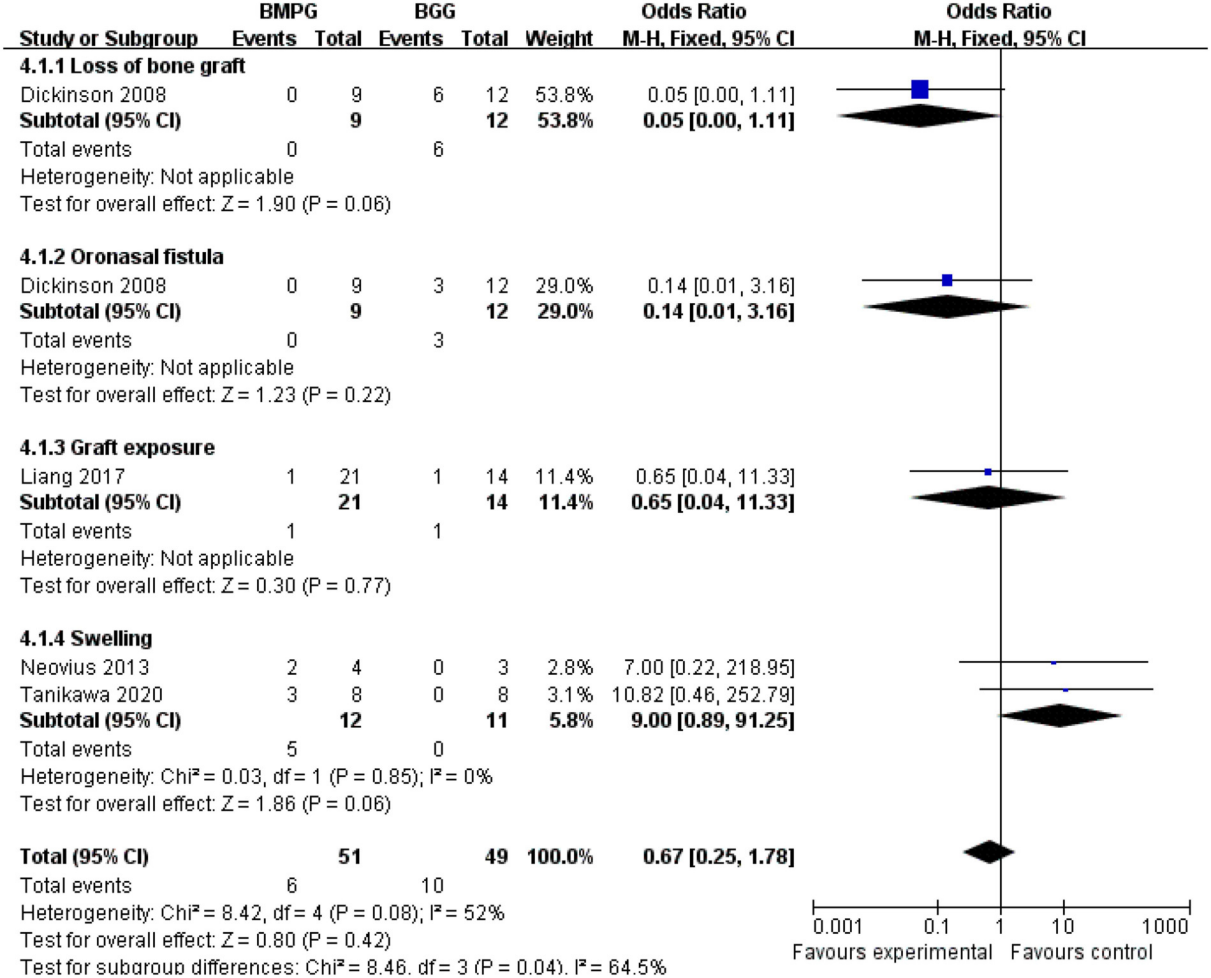
Forest plot of complications after surgery.

All the included studies reported postoperative complications, but only Dickinson et al., Liang et al., Neovius et al., and Tanigawa et al. had a patient who had an eventful postoperative course. A fixed-effects model was used to analyze the pooled data. There were no statistically significant differences in complications between the patients who received autologous bone grafting and those who received rhBMP-2 (MD 0.67; 95% CI 0.25–1.78; *p* = 0.42) (Figure 8). Finally, a sensitivity analysis was carried out to check the rationality of the system. Changing from an OR value to an relative risk (RR) value changes the results. Although the meta-analysis showed no difference in complications between autologous bone grafting and rhBMP-2, this result must be treated with caution.

### Risk of bias across studies

The quality of evidence, rated using the GRADE tool, was low, implying moderate confidence in the estimated effect from the analyzed outcomes. The main reason for the poor quality of evidence was an “unclear” risk of bias among the included studies (Figure 2). Due to the small number of studies included, publication bias was not examined.

## Discussion

### Summary of evidence

This study included six RCTs (21–26) that focused on the safety and efficacy of rhBMP-2 in patients with a cleft lip and palate after 3, 6, and 12 months of follow-up. During the 6-month observation period, autologous bone grafting showed a superior effect on the bone formation rate compared to alveolar repair with rhBMP-2, and this difference was statistically significant. This is one of the most important findings of this study, but it is worth noting that this difference disappeared at 12 months. However, a closer examination revealed that autologous bone grafting was still superior to rhBMP-2 at 3 and 12 months, even if the results showed no statistically significant difference between the two groups. This interesting phenomenon is caused by several factors. First, during the 3-month follow-up period, the jaw segment was slightly clearer after bone grafting, and because of this, the difference in osteogenesis was very small. Second, at 12 months, the difference between the two groups disappeared, but unlike the 3-month follow-up, this may be due to the longer time after bone grafting, as the osteogenesis rate gradually decreases and total osteogenesis will be close to the defect volume, resulting in the disappearance of the difference. It is worth mentioning that the 4-month follow-up results from Herford et al. were included in the 3-month follow-up data. This is because Herford et al. used Honma et al.’s measurement method (27), starting with a continuous 1 mm section, which is consistent with the CT scans used by Canan et al. The accuracy and quality of multichannel helical CT scans using 1 mm sections are high (21, 28). Moreover, Balaji et al. (29) argue that, considering the cost and, most importantly, considering radiography, it is still a good method to study the efficiency of bone formation in the fourth month after surgery (30), which is consistent with Honma et al.'s views. Therefore, the results of Herford et al. were included in the 3-month follow-up data.

In the bone formation volume analysis, only the study of Canan et al. was included, and the results of the two groups in the study were not significantly different. Kim et al. (2) indicate that in patients without lateral incisor tooth germs, early secondary ABG should be avoided before the canine eruption, as bone resorption due to disuse will be inevitable. Canine tooth eruption generally occurs before the age of 12 years, but the patients included in Canan et al.’s study were 8–15 years old, and some patients inevitably experienced strong bone resorption in the first stage of autologous bone grafting (21, 31). Furthermore, the bone induction capacity of rhBMP-2 declines after 6 months (21), which may be the reason for this result. It is worth noting that the pooled data indicate that autologous bone grafting resulted in statistically significantly higher bone volume.

The same applied to the bone mineral density analysis, and although the results suggest higher osteogenic density in the rhBMP-2 group after 6 months, this result seems inevitable given the significant bone resorption experienced by those who underwent autologous bone grafting. Because only one study was included in the bone mineral density analysis and the sample size was small, a sensitivity analysis was performed. The pooled data results changed and a random-effects model was used, but the results were not statistically significant.

The presence of postoperative complications following autologous bone grafting vs. rhBMP-2 grafting is another important finding. These included loss of bone graft, an oronasal fistula, graft exposure, and, most commonly, swelling. Contrary to the expected results, no significant differences between the two groups were found. Dickinson et al. argue that the use of rhBMP-2 reduces postoperative complications compared with a traditional iliac bone graft, reporting three patients with oronasal fistula and six patients with loss of bone graft in the rhBMP-2 group. However, contrary to expectations, the difference between the two groups was not statistically significant. Tanikawa et al. did not report any loss of bone graft, which is consistent with our results. As for postoperative donor pain and residual scarring in the donor area, this is inevitable, and it is also the most controversial side effect of autologous iliac bone grafting (2, 5). The primary clinical driver for the use of rhBMP-2 is the elimination of iliac crest donor site pain and scarring. However, postoperative pain is often overestimated, and conventional analgesic medications can achieve good results (32). Moreover, by using a trephine to core-drill a sample, postoperative pain can be reduced (5). In fact, this complication is difficult to evaluate because it is intrinsically linked to each surgeon's surgical habits and surgical methods.

Regarding postoperative swelling, Herford et al. and Liang et al. mentioned this outcome, but did not provide statistics. Thus, only Tanikawa et al. was included, and while Alonso et al. (33) used the same concentration, no complications related to swelling were reported. Dickinson et al. and Canan et al. did not report any adverse effects in the treatment groups. Local inflammation induced by rhBMP-2 has been shown to be the main cause of postoperative swelling (14, 34, 35), and patients in pooled studies are unlikely to be free of any adverse effects, which is likely to lead to false-negative results. Numerous studies have clearly shown that postoperative swelling is the most significant side effect (14), especially in the pediatric population, and occasionally requires pharmacological intervention, such as cortisol therapy or even secondary surgery to remove the rhBMP-2 implant (36). However, swelling is still easily ignored and can lead to a series of problems and even serious consequences. For example, Ramly et al. reported that more than half of their patients had severe edema during evaluation and 4.6% of these had wound dehiscence due to edema (37). If edema duration exceeds 2 weeks, the frequency and severity of rhBMP-2 exposure is greatly increased (14). de Freitas et al. (38) argued that this greatly increases the recovery time after surgery, and the edema makes it impossible to use a temporary prosthesis for 2 weeks after surgery. Ramly et al. also reported a patient who underwent craniofacial surgery, which caused airway swelling due to exposure to rh-BMP2, after which prolonged intubation was required. Coincidentally, the FDA (Food and Drug Administration) issued a warning about BMP-2, indicating that swelling of the throat and neck after administering rhBMP-2 may be life-threatening.

Notably, among the included studies, five used collagen sponges as the rhBMP-2 carrier, while one utilized hydrogel. Given that collagen sponges and hydrogels exhibit different growth factor release kinetics, this variation in carrier type may have potentially affected the bone regeneration efficacy and postoperative complication rates. Differences in degradation rate, protein release pattern, and local microenvironment induced by different carriers could also be a primary source of heterogeneity in the pooled results.

While rhBMP-2 was approved as an alternative to autologous bone grafting for sinus and local alveolar ridge augmentation in 2007 (14), complications other than side effects, such as ectopic bone formation, carcinogenicity, slow recovery of chewing function for soft or general foods, and effects on normal tooth eruption and later orthodontic treatment, have been catastrophic for patients (13, 29). Currently, the use of rh-BMP2 in children lacks safety and long-term efficacy data; therefore, the use is still off-label (2).

RhBMP-2 is currently most commonly used in spine surgery, and after being approved by the FDA and initially thought to be safe, a series of associated complications has been reported. These complications include ectopic bone formation, bone resorption or remodeling, hematoma, inflammatory swelling, radiculitis, cystoid osteolysis, retrograde ejaculation, compartment syndrome, cerebrospinal fluid leakage, tumors, and neck swelling (13, 39–43). There is currently much controversy about tumors, and Kelly et al. (44) concluded in a 2.9-year study that rhBMP-2 was not associated with an increased risk of various cancers. However, Katsuno et al. (45) report that the aggressiveness of breast cancer may be increased through the SMAD pathway. It is important to note that although it is not thought to increase cancer risk, many cancers have BMP receptors; thus, stimulating the receptors leads to metastasis and growth. Major complications due to rhBMP-2 use in orthopedics have been reported so far, and some have also been reported in the reconstruction of alveolar clefts; thus, caution should be exercised when weighing the benefits against the risks before using the product in patients. In patients undergoing alveolar bifida reconstruction, there is a higher inflammatory response and dependence on the location within the oral cavity, particularly in younger patients.

This study has several limitations. First, the primary limitation of this meta-analysis is the small number of included RCTs (only six studies) and the potential presence of multiple types of bias; thus, the conclusions should be interpreted with caution. Moreover, the included randomized controlled trials had relatively small sample sizes. In particular, Herford et al. only enrolled 12 patients in total, among whom 10 received rhBMP-2 and 2 were treated with autologous bone grafting. In addition, the limited sample size of the enrolled studies led to low statistical power to detect rare but serious adverse events and complications. Accordingly, we cannot exclude the possibility that some uncommon severe complications related to rhBMP-2 were missed in our pooled analysis. This inadequate statistical power may explain why no significant intergroup difference in overall complication rate was observed in our analysis, even though clinical concerns persist regarding the higher potential complication risk of rhBMP-2. To enable evidence-based, standardized recommendations for optimal bone grafting techniques in patients with alveolar clefts, well-designed, multicenter, prospective RCTs with consistent treatment protocols and larger sample sizes are required. Further large-sample, multicenter studies with longer follow-ups are also warranted to comprehensively assess the long-term safety and rare complication profile of rhBMP-2.

Second, regarding clinical heterogeneity, all the randomized controlled studies included in our meta-analysis did not use the same concentrations and doses of rhBMP-2. The concentration used in Herford et al. was 1.0 mg/mL, and the rest of the et al. studies used 1.5 mg/mL. The dosages varied widely, including 2.1, 3.2–4.2, and 4.2 mg. At present, the therapeutic concentration of rhBMP-2 is still controversial, and no dose guidelines have been established (46, 47). The most commonly used dose of rhBMP-2 is 1.5 mg/mL; however, Li et al. (48) found that the application of this dose in alveolar reconstruction in patients with CLP did not achieve any positive results. RhBMP-2 treatment has been confirmed in animal models to be dose-dependent. Moreover, in Neovius et al., rhBMP-2 doses of 0.05 and 0.25 mg/mL did not produce a positive therapeutic effect, requiring higher doses. However, the problems that come with high doses are obvious, as the inflammatory response caused by high concentrations and high doses is greater and the resulting complications, such as swelling, caused by inflammatory reactions, will become uncontrollable. Therefore, there is a need to further clarify the concentration and dose effects and to clarify the indications and dosage of rhBMP-2 so that clinicians can make appropriate decisions.

Third, regarding methodological heterogeneity due to between-group baseline comparisons, allocation concealment, and sequence generation, none of the six included RCTs described sequence generation, although they all claimed that all the groups were randomly assigned, which may have introduced some selection bias.

Finally, although the carrier of BMP-2 has not been the focus of published randomized controlled trials, it is well-established that the carrier exerts a critical influence on the release kinetics and biological activity of BMP-2 in both *in vitro* and *in vivo* settings. Inappropriate carrier selection may result in uncontrolled release profiles and increase the risk of adverse events. For instance, collagen sponges, the conventional carrier of BMP-2, are prone to inducing burst release, leading to excessively high local concentrations of BMP-2 in the early phase. Such rapid and unregulated release not only shortens the effective duration of osteogenic induction but also elevates the risk of soft tissue swelling, inflammatory responses, and even ectopic bone formation. Therefore, carrier characteristics represent important confounding factors that may affect the therapeutic efficacy and safety of BMP-2. Future clinical investigations should take carrier type, release kinetics, and biocompatibility into account to optimize the osteogenic efficiency and minimize the potential risk of BMP-2-based bone regeneration procedures.

Overall, more RCTs are needed to examine whether rhBMP-2 improves the effects of autologous bone regeneration in alveolar cleft grafts, based on the evidence and limitations presented in this review. To minimize bias in RCTs, the following design is recommended: use of a standardized concentration dose and surgical procedures, rigorous patient recruitment, use of correct randomization methods and appropriate allocation concealment, blinding of patients and outcome assessors, and correction of measurements.

## Conclusion

In this study, there was no significant difference between autologous bone grafts and rhBMP-2 grafts in the overall assessment of osteogenesis, indicating that rhBMP-2 has no significant clinical advantage over autologous bone grafts in alveolar fissure reconstruction. Furthermore, although no statistical differences were found in this cohort in terms of complications, the clinical literature suggests that rhBMP-2 may cause more postoperative complications, such as postoperative swelling. Thus, further clinical research is required.

## Data Availability

The original contributions presented in the study are included in the article/Supplementary Material, further inquiries can be directed to the corresponding author.
